# Miscellaneous

**Published:** 1888-05

**Authors:** 


### MISCELLANEOUS.

Rio Chemical Co's Preparation.—We hear the best accounts of the efficacy of the "Aletres Cordial and other preparations of the Rio Chemical Company of St. Louis advertised in this Journal.

Macadam Grigor, L.R.C.S., L.R.C.P. Alexandra Avenue, Battersea Park, London, says: F. O., widow, 32 years of age, one child, suffered for years, and was frequently under medical treatment, getting little or no relief. When she came under my care, about three months ago, I found her very weak and anæmic, complained of pain in left hypogastric region and sympathetic vomiting. Under treatment she improved in general health, but still the menorrhagia and leucorrhea continued, though I had exhausted the remedies used in such cases. When the Aletris Cordial came under my notice about six months ago, I put my patient under its treatment, with the result the menorrhea and leucorrhea have ceased, and the slight prolapsus uteri gives no discomfort. I may state that I still keep her under the tonic.

A new lease of life will surely result from the use of WINCHESTER'S HYPOPHOSPHITE OF LIME AND SODA, acknowledged to be the most perfect Tonic ever discovered. It will rapidly build up, invigorate and vitalize the entire system. It will restore the appetite, strengthen the organs of digestion, enrich or renovate and replenish the blood. It will invigorate the brain and nervous system, and speedily and permanently cure general debility, nervous prostration, weakness, etc., in fact all afflictions caused by impaired vigor of the brain and loss of nervous power and energy.

From actual experiment we feel justified in recommending the self-raising "Imperoyal Wheat and Buckwheat Flour," manufactured and sold by M. D. Stevens, room 18, Mercantile Exchange as equal if not superior to any other similarly prepared bread stuffs in the market. Try it.

Dr. T. D. Crothers, a very high authority, says: "It is a fact beyond question, that alcohol in excess produces changes of brain circulation and nutrition, also vaso-motor paralysis, with congestion and derangement of the heart's action and diminution in the quality of the blood. The result is that both brain and nerve functions are impaired and the capacity to realize the nature and consequence of conduct and thoughts is lessened—the victim is actually and literally on the road to insanity. The inebriate cannot be sane, for his brain is physiologically and pathologically changed.

A narrow pointed red tipped tongue with mucous membrane of the mouth of the same color, indicates that the blood needs an acid, while a broad, pallid tongue covered with a white pasty fur along with pale membrane of the mouth, indicates that the blood is already too acid and that an alkali is needed.

"I regard the use of beer as the true temperance principle. When I work all day and am exhausted, nothing helps me like a glass of beer. It assists nature you understand," said Remson to Benson. "It makes a fool of me," Benson replied. "Just so," exclaimed Remson; "that's what I say; it assists nature."

Regulate the Regulator with Warner's Log Cabin Sarsaparilla. Manufactured by proprietors of Warner's Safe Cure. Largest bottle in the market. All druggists sell it.

Is Consumption Contagious?—After the study of nearly twelve thousand cases, Dr. Hermann Brehmer, an able German physician, rejects the theory of the contagiousness of pulmonary consumption. He finds the disease to be due to deficient nutrition of the lung, which may result from many causes. He believes that the operation of all the causes may produce such changes that it may be possible, years in advance, to predict with great probability which members of a family will be afflicted with pulmonary consumption, and which will remain healthy.—*Medical Herald.*

A novel aid to instantaneous photography has been devised by two Berlin chemists. It consists of an explosive powder made of pulverized magnesium, chlorate of potash, and sulphide of antimony. When this is ignited it will illuminate the darkest room with a flash lasting only one-fortieth of a second, but long enough to obtain a photograph of a person or object in the apartment.—*Am. Druggist.*

How to Cure Warts.—Place the thumb upon the wart and press it against the bone. Move the wart backwards and forwards upon the bone until the roots become irritated or sore, when the wart will disappear. So says a correspondent of the *Scientific American.*

### HE AND I.

Yes, I knew him when he came,
Young in hope and high in aim
As a thrifty pine; from Maine.

In his cheeks were roses, then,
And his eyes, so clear and bright,
Sparkled with an eager light
That was never wanting when
Buckeye maids with winsome wile
Sought the favor of his smile.

Yes, I knew him years ago,
Where Ohio's waters flow,
And to-day, we met again
At the bridges farther span,
And I marveled if't could be
That this old and grizziy wight,
Bent and bald and dim of sight,
Were my old-time friend, H. P.
And in turn, most likely, he
Queried just the same of *me.*

